# Retracted: Hepatocellular Carcinoma: Novel Molecular Targets in Carcinogenesis for Future Therapies

**DOI:** 10.1155/2018/4560161

**Published:** 2018-04-30

**Authors:** BioMed Research International

BioMed Research International has retracted the article titled “Hepatocellular Carcinoma: Novel Molecular Targets in Carcinogenesis for Future Therapies” [1]. The first author, Gaetano Bertino, accepts full responsibility and apologizes to the journal and the other authors. The author list has been corrected to remove Salvatore Gruttadauria, Isidoro Di Carlo, and Adriana Toro, who were not involved in the preparation or submission of this article and should not have been listed as authors. The other authors were not involved in the preparation of the article content and are not culpable in this plagiarism and are victims of this action.

Much of the article was previously published as follows:

1. G. Bertino, S. Demma, N. Bertino, and A. Ardiri, “Management of hepatocellular carcinoma: an update at the start of 2014,” Journal of Gastrointestinal & Digestive System, vol. 4, no. 2, pp. 1–7, 2014. doi:10.4172/2161-069X.1000178
2. G. Bertino, I. Di Carlo, A. Ardiri et al., “Systemic therapies in hepatocellular carcinoma: present and future,” Future Oncology, vol. 9, no. 10, pp. 1533–1548, 2013. doi:10.2217/fon.13.171

The article was also found to contain a substantial amount of material from the following published articles:

(3) E. Breous and R. Thimme, “Potential of immunotherapy for hepatocellular carcinoma,” Journal of Hepatology, vol. 54, no. 4, pp. 830–834, 2011. doi:10.1016/j.jhep.2010.10.013
(4) J. M. Llovet, and J. Bruix, “Molecular targeted therapies in hepatocellular carcinoma,” Hepatology, vol. 48, pp. 1312–1327, 2008. doi:10.1002/hep.22506

Figure 2 in the article is reproduced from Figure 1 in Breous and Thimme without attribution.
